# Rice *DEP1*, encoding a highly cysteine-rich G protein γ subunit, confers cadmium tolerance on yeast cells and plants

**DOI:** 10.1093/jxb/ert267

**Published:** 2013-10-25

**Authors:** Shuta Kunihiro, Tatsuhiko Saito, Taiki Matsuda, Masataka Inoue, Masato Kuramata, Fumio Taguchi-Shiobara, Shohab Youssefian, Thomas Berberich, Tomonobu Kusano

**Affiliations:** ^1^Graduate School of Life Sciences, Tohoku University, 2-1-1 Katahira, Aoba, Sendai, Miyagi 980-8577, Japan; ^2^National Institute of Agrobiological Sciences, Tsukuba, Ibaraki 305-8602, Japan; ^3^Faculty of Bioresource Sciences, Akita Prefectural University, 241-7 Kaidobata Nishi, Akita 010-1095, Japan; ^4^Biodiversity and Climate Research Center (BiK-F), D-60323 Frankfurt, Germany

**Keywords:** cadmium tolerance, cysteine-rich protein, G protein γ subunit, heterotrimeric G protein signalling, Oryza sativa, OsDEP1.

## Abstract

A rice cDNA, *OsDEP1*, encoding a highly cysteine (Cys)-rich G protein γ subunit, was initially identified as it conferred cadmium (Cd) tolerance on yeast cells. Of the 426 aa constituting OsDEP1, 120 are Cys residues (28.2%), of which 88 are clustered in the C-terminal half region (aa 170–426). To evaluate the independent effects of these two regions, two truncated versions of the *OsDEP1*-expressing plasmids pOsDEP1(1–169) and pOsDEP1(170–426) were used to examine their effects on yeast Cd tolerance. Although OsDEP1(170–426) conferred a similar level of Cd tolerance as the intact OsDEP1, OsDEP1(1–169) provided no such tolerance, indicating that the tolerance effect is localized to the aa 170–426 C-terminal peptide region. The Cd responses of transgenic *Arabidopsis* plants constitutively expressing *OsDEP1*, *OsDEP1(1–169)* or *OsDEP1(170–426)*, were similar to the observations in yeast cells, with *OsDEP1* and *OsDEP1(170–426)* transgenic plants displaying Cd tolerance but *OsDEP1(1–169)* plants showing no such tolerance. In addition, a positive correlation between the transcript levels of *OsDEP1* or *OsDEP1(170–426)* in the transgenics and the Cd content of these plants upon Cd application was observed. As several *Arabidopsis* loss-of-function heterotrimeric G protein β and γ subunit gene mutants did not show differences in their Cd sensitivity compared with wild-type plants, we propose that the Cys-rich region of OsDEP1 may function directly as a trap for Cd ions.

## Introduction

Cadmium (Cd) is one of the transition metals that is non-essential for almost all living organisms. It is also a noxious compound that inactivates and denatures structural and functional proteins of organisms by binding to free sulfhydryl groups, thereby inhibiting their growth and development. Another aspect of Cd toxicity is derived from its chemical similarity to metal co-factors or coordinated metals, such as Zn, Fe, and Ca, of enzymes, signalling intermediates, and transcription factors, especially the zinc-finger type (DalCorso *et al.*, 2008; Verbruggen *et al.*, 2009). Due to its high toxicity, Cd pollution poses serious problems both to our natural environment and to human health (Järup, 2003). To address such levels of Cd contamination, phytoremediation is being promoted as a promising approach, especially as it is more environmentally friendly and imposes a smaller financial burden than alternative physico-chemical approaches. Towards establishing easy-to-apply phytoremediation strategies, it is essential to identify important factors in the plant genome that can be exploited for such approaches.

To cope with Cd toxicity effects, plants are known to be equipped with the potential to chelate and extrude Cd, to sequester Cd into vacuoles, and to dissipate reactive oxygen species triggered by Cd. For the chelation of heavy metals, including Cd, various cysteine (Cys)-rich proteins are employed by plants. Small Cys-rich peptides, called metallothioneins (MTs), are the major chelators of Cd (Ecker *et al.*, 1986; Freisinger, 2008). Recently, the class A heat-shock transcription factor HsfA4a was identified as a wheat clone that confers strong Cd tolerance in yeast and rice by upregulating the *MT* gene (Shim *et al.*, 2010). Several other Cys-rich proteins, of various sizes and cellular localizations, have also been found to provide Cd tolerance (Willuhn *et al.*, 1994; Song *et al.*, 2004; Kuramata *et al.*, 2009; Matsuda *et al.*, 2009).

For Cd extrusion, AtPDR8, an ATP-binding cassette (ABC)-type transporter, has been found to be involved in Cd efflux at the plasma membrane (Kim *et al.*, 2007). In addition, the wheat *TM20* gene, which encodes a hydrophobic protein with 20 transmembrane domains, has been shown to stimulate Cd efflux when overexpressed in yeast cells, suggesting that TM20 functions to pump out Cd by an unknown mechanism (Kim *et al.*, 2008). Furthermore, yeast cells and *Arabidopsis* plants constitutively expressing the *Digitaria ciliaris CDT1* gene have been found to contain less than half the Cd levels of their respective controls, indicative of a reduced accumulation of Cd in these organisms, most probably through Cd extrusion (Kuramata *et al.*, 2009). Finally, it is worth noting that tobacco plants grown in Cd-containing medium were found to secrete Cd-containing amorphous materials through the tips of their long trichomes, demonstrating the role that trichomes play in Cd extrusion (Choi *et al.*, 2001).

For Cd sequestration to vacuoles, the *Saccharomyces cerevisiae* YCF1 (yeast cadmium factor 1), a vacuolar-localized ABC-type transporter, is known to function in the sequestration of both glutathione (GSH) conjugates and (GSH)_2_–Cd complexes into the vacuole; indeed, deletion of this gene (*YCF1*) renders the host yeast cells Cd hypersensitive (Li *et al.*, 1996). Similarly in plants, two ABC-type vacuolar transporters that mediate arsenic and Cd tolerance have recently been identified in *Arabidopsis* (Mendoza-Cózatl *et al.*, 2010; Song *et al.*, 2010). P_1B_-ATPase subfamily members in both *Arabidopsis* (AtHMA3) and rice (OsHMA3) have been shown to be localized to the vacuolar membrane (tonoplast) and to be essential for the sequestration of Cd into vacuoles (Morel *et al.*, 2009; Ueno *et al.*, 2010; Miyadate *et al.*, 2011). Tonoplast membrane-localized Cd^2+^/proton antiporters, such as AtCAX2 and AtCAX4, have also been shown to transport Cd without modification into vacuoles (Korenkov *et al.*, 2007).

Finally, Cd is known to induce oxidative damage, such as through lipid peroxidation, which can lead to changes in membrane functionality and protein carbonylation (Romero-Puertas *et al.*, 2002, 2004). Antioxidants and antioxidant-synthesizing enzymes have been implicated in the enhanced tolerance of plants to Cd toxicity (DalCorso *et al.*, 2008; Verbruggen *et al.*, 2009).

The rice *DEP1* (*DENSE AND ERECT PANICLE 1*) locus was first identified by two independent research groups with quantitative trait loci analysis to control grain yield, grain numbers per panicle, and panicle morphology (Huang *et al.*, 2009; Zhou *et al.*, 2009). Deletion of the *DEP1* gene during rice domestication was proposed to enhance meristematic activity and result in reduced inflorescence internode lengths that thereby increased grain numbers per panicle and, consequently, grain yields (Huang *et al.*, 2009; Zhou *et al.*, 2009; Taguchi-Shiobara *et al.*, 2011). Recently, *Arabidopsis* AGG3, a DEP1 homologue, was identified as an *Arabidopsis* heterotrimeric GTP-binding protein (G protein) γ subunit (Chakravorty *et al.*, 2011; Thung *et al.*, 2012). Unlike the complex mammalian system, *Arabidopsis* has only one α (GPA1), one β (AGB1), and three γ (AGG1, AGG2, and AGG3) subunits as components of the heterotrimeric G protein system (Botella 2012; Thung *et al.*, 2012). So far, AGG3 has been shown to be involved in both guard cell K^+^-channel regulation and morphological development (Chakravorty *et al.*, 2011; Li *et al.*, 2012*a*,*b*).

Here, we identified *OsDEP1* as a cDNA clone that confers Cd tolerance to yeast cells. The gene product, OsDEP1, is highly Cys-rich and is a component of the heterotrimeric G protein signalling pathway (Botella, 2012). Based on the results obtained, we discuss a functional role for this G protein subunit in the Cd stress response.

## Materials and methods

### Plant materials

Rice plants (*Oryza sativa* cv. Nipponbare) were grown hydroponically in 40% strength Hoagland’s solution #2 [2mM Ca(NO_3_)_2_.4H_2_O, 2mM KNO_3_, 0.8 μM MgSO_4_.7H_2_O, 0.0002% FeSO_4_.EDTA] or in soil in a greenhouse. Seeds of *Arabidopsis thaliana* ecotype Columbia (Col-0) and the loss-of-function heterotrimeric G protein gene mutants of Columbia background (*gpa1-4*, *agb1-1*, *agb1-2*, *agg1-1C*, *agg3-1* and the triple mutant *agg1-1C agg2-1 agg3-1*), kindly provided by Professor J. Botella (Botella, 2012), were germinated and grown on vermiculite in a growth chamber at 22 °C under a 16h light/8h dark photocycle.

### Yeast strains

The yeast strains used in this study were obtained from EUROSCARF (http://web.uni-frankfurt.de/fb15/mikro/euroscarf/index.html), Frankfurt, Germany. The wild-type (WT) strain used was *S. cerevisiae* BY4742 with the relevant genotype (*MATα*; *his3Δ1*, *leu2Δ0*, *lys2Δ0*, *ura3Δ0*). To identify cDNA clones conferring Cd tolerance on yeast cells, the Cd-sensitive BY4742 *Δycf1* mutant (*MATα*; *his3Δ1*, *leu2Δ0*, *lys2Δ0*, *ura3Δ0*; *YDR135c::kanMX4*) was employed. In addition, *Δcup2* (*MATα*, *his3Δ1*, *leu2Δ0*, *lys2Δ0*, *ura3Δ0*, *YGL166w::kanMX4*) was used to test for Cu^2+^ tolerance. See Supplementary Methods at *JXB* online for details of the other metal-sensitive strains used.

### Preparation of an *O. sativa* cDNA library

*O. sativa* cv. Nipponbare seedlings were grown in Murashige–Skoog medium (Murashige and Skoog, 1962) for 2 weeks. Total RNA was isolated from fresh rice seedlings by an SDS/phenol method (Shirzadegan *et al.*, 1991), and mRNA was subsequently purified using an mRNA purification kit (QuickPrep mRNA Purification kit; GE Healthcare, Milwaukee, USA). The mRNA fraction was then converted to cDNA using a SMART cDNA library construction kit (BD Bioscience Clontech, Palo Alto, CA, USA). After *Sfi*I enzyme digestion, the resulting cDNA was ligated into the *Sfi*IA and *Sfi*IB sites of a modified yeast expression vector, termed pGK1, of p112A1NE (Riesmeier *et al.*, 1992), yielding a *O. sativa* cDNA library.

### Isolation of Cd-tolerant clones from the rice cDNA library

To isolate Cd-tolerant clones from the rice cDNA library, we introduced the library into the yeast *Δycf1* mutant cells using the lithium acetate method (Ito *et al.*, 1983). Yeast colonies that grew on medium containing 20–60 μM CdCl_2_ were selected and their plasmids isolated, and these plasmids were then re-introduced into *Δycf1* cells to reconfirm the Cd tolerance of the clones.

### Construction of pOsDEP1 and its two derivatives

The fragment covering the ORF of *OsDEP1* (Os09g0441900) was amplified using the primer pair Os09g0441900-F (5′-TTAGGCCATTACGGCCGTGAAGGCGGCGAGGGT-3′; the underlined sequence denotes the *Sfi*IA restriction site in this study) and Os09g0441900-R (5′-TTAGGCCGAGGCGGCCTCAA CATAAGCAACCAC-3′, the underlined sequence denoting the *Sfi*IB site in this study). The sequence-verified fragment was inserted into the *Sfi*IA and *Sfi*IB restriction sites of the yeast expression vector, pGK1, resulting in construct pOsDEP1. The *OsDEP1* gene encodes a 426 aa residue protein that consists of two domains (Huang *et al.*, 2009; Zhou *et al.*, 2009; Taguchi-Shiobara *et al.*, 2011): an N-terminal half (aa 1–169) and a Cys-rich C-terminal half (aa 170–426). Thus, two *OsDEP1*-derived fragments, covering the OsDEP1(1–169) and OsDEP1(170–426) regions, were amplified using the following primer pairs, respectively: Os09g0441900-F and OsDEP1(1–169)-R (5′-TTAGGCCGAGGCGGCCTCAGTTCGGTTTGCAG-3′), and OsDEP1(170–426)-F (5′-TTAGGCCATTACGGCCATGTGC TGTAAACCTAACTGCAG-3′) and Os09g0441900-R. The sequence-verified fragments were subcloned into the *Sfi*IA and *Sfi*IB sites of pGK1 vector, resulting in the respective constructs pOsDEP1(1–169) and pOsDEP1(170–426).

### Cd and Cu tolerance assays in agar medium and in liquid culture

*S. cerevisiae* BY4742 *Δycf1* or *Δcup2* strain was transformed with either the pGK1 empty vector (EV), or with pOsDEP1, pOsDEP1(1–169), or pOsDEP1(170–426). The *S. cerevisiae* WT BY4742 strain transformed with EV was also used as a control. The turbidity of the SD-Ura (synthetic drop-out lacking uracil) liquid cultures, inoculated with the respective transformants, was adjusted to an optical density at 600nm (OD_600_) of 1.0, and tenfold serial dilutions then prepared aseptically. Subsequently, 5 μl of each of the dilution series was spotted onto SD-Ura agar medium with or without 50 μM CdCl_2_ or 300 μM CuCl_2_, and incubated at 30 °C for 3 d. In addition, growth of the yeast transformants described above in SD-Ura liquid medium supplemented with 40 μM CdCl_2_ was monitored at OD_600_.

### Generation of transgenic *Arabidopsis* plants

As the *OsDEP1-*coding region contained a single *Sac*I site, we first removed this *Sac*I site from the *OsDEP1*-coding region, without changing the amino acid sequence, by two-step PCR using *OsDEP1* cDNA template, KOD-Plus DNA polymerase (Toyobo, Japan) and the following primer pairs. The first PCR was performed with two primer pairs, DEP-ox-F (5′- CGGTCTAGACAAGGAGATATAAC AATGGGGGAGGAGGCGGT-3′; the new *Xba*I site is underlined and the start codon is italic) and dep1-rr (5′- CGGGCTGCGCTCCTT CAAGGAAGT-3′, mutated site is underlined), and dep1-ff (5′-AAGGAGC*G*CAGCCCGTTTCTCGTT-3′; mutated site is underlined) and DFP1-ox-R (5′-CAGGAGCTCTCAACATAA GCAACCACT-3′; the new *Sac*I site is underlined and the stop codon is italic). The second PCR was then performed on mixtures of the first PCR products using the primer pair DEP-ox-Fw and DEP-ox-R. In addition to the OsDEP1 (aa 1–426, *Sac*I site mutated) fragment, the OsDEP1(1–169) and OsDEP1(170–426) fragments were also amplified with the following primer pairs; DEP1-ox-F and OsDEP1lackCys-rich-ox-R (5′-CAGGAG CTCTCAGTTCGGTTTGCAGCA-3′; *Sac*I site is underlined), and OsDEP1Cys-rich-ox-F (5′-CGGTCTAGACAAGGAGATAT AACAATGTGCTGTAAACCTAA-3′; *Xba*I site is underlined) and DEP1-ox-R, respectively. The sequence-verified fragments encompassing the complete *OsDEP1* ORF and OsDEP1(1–169) and OsDEP1(170–426) were digested with *Xba*I and *Sac*I, and subcloned into the respective restriction sites of the binary vector, pBI121 (Clontech), yielding pBI121OsDEP1, pBI121OsDEP1(1–169), and pBI121OsDEP1(170–426), respectively. These plasmids were introduced by the freeze–thaw method into *Agrobacterium tumefaciens* GV3101 cells (Koncz and Schell, 1986), which were then used to transform *A. thaliana* ecotype Col-0 plants by the floral dip method (Clough and Bent, 1998). Transformants were selected on Murashige–Skoog agar medium containing 50mg ml^–1^ of kanamycin (Km) and 50mg ml^–1^ of carbenicillin. T_2_ seeds obtained from self-fertilization of primary transformants were surface sterilized and grown on Km plates. Lines showing a 3:1 (resistant:sensitive) segregation ratio were selected and used to produce homozygous (Km^R^/Km^R^) T_3_ lines that were used for further study.

### Reverse transcription-PCR (RT-PCR) analysis

Expression analysis of the transgene in transgenic *Arabidopsis* plants was performed by RT-PCR. Total RNA was extracted from whole seedlings, reverse transcribed, and then semi-quantitatively amplified using the following primer pairs: *OsDEP1* forward, (5′-GTGAAGGCG GCGAGGGT-3′) and *OsDEP1* reverse (5′-TCAACATAAG CAACCAC-3′); *OsDEP1(1–169)* forward, the same forward primer used for *OsDEP1*, and *OsDEP1(1–169)* reverse (5′-TCAGTTCG GTTTGCAG-3′); *OsDEP1(170–426)* forward (5′-ATGTGCTGTAA ACCTAACTGCAG-3′) and *OsDEP1(170–426)* reverse, the same reverse primer used for *OsDEP1*. As a control, the *Arabidopsis tubulin* gene was amplified using the following primers pair: *tubulin* forward (5′-CGTGGATCACAGCAATACAGAGCC-3′) and *tubulin* reverse (5′-CCTCCTGCACTTCCACTTCGTCTTC-3′).

### Response of transgenic plants to CdCl_2_ and measurement of Cd contents

The seeds of control transgenics transformed with pBI121 (Clontech), and transgenics expressing full-length *OsDEP1* or the *OsDEP1(1–169)* and *OsDEP1(170–426)* derivatives were surface sterilized, rinsed, and placed onto MRGL medium (Fujiwara *et al.*, 1992; Kuramata *et al.*, 2009) solidified with 1% (w/v) gellan gum either without CdCl_2_ (control) or with appropriate concentrations of CdCl_2_. After 2 weeks of incubation, seedling growth was analysed and root lengths were quantified using ImageJ software (National Institutes of Health, http://rsbweb.nih.gov/ij/). Five-d-old *Arabidopsis* seedlings grown on MRGL medium were transferred to deionized water containing 5 μM CdCl_2_ and incubated for a further 5 d. The seedlings (*n*=5) were washed thoroughly with sterilized water, blotted with a paper towel, and then dried at 65 °C for 1 d. Dried plant samples were digested with 60% nitric acid at 60 ºC, and the Cd contents were analysed by Zeeman atomic absorption spectrometry (AA240Z; Varian).

### Statistical analysis

Data analysis was performed using the statistical tools (Student’s *t*-test) of Microsoft Excel software.

## Results

### Identification of *OsDEP1* as a clone conferring Cd tolerance to yeast cells

Of the approximately 3.0×10^5^ *O. sativa* cDNA clones analysed, six were identified as conferring Cd tolerance. Of these clones, three encoded class I MTs; the fourth clone was a homologue of *Hypochaeris radicata HrCDT3* (GenBank accession no. AB454513) that has been implicated in Cd tolerance (published only in the NCBI database); the fifth clone encoded *OsCDT1* (GenBank accession no. AK121052), which was also previously identified as a Cd tolerance-related clone (Kuramata *et al.*, 2009; Matsuda *et al.*, 2009); and the sixth clone was found to encode a protein that showed a high level of similarity to a keratin-associated protein (GenBank accession no. FJ039905). Interestingly, the gene corresponding to this latter clone was first identified as the causal gene of the rice panicle morphology mutant by two independent research groups (Huang *et al.*, 2009; Zhou *et al.*, 2009) and was thus termed *OsDEP1* (*O. sativa DENSE AND ERECT PANICLE 1*). More importantly, a recent study has indicated that the gene product, OsDEP1, is an isoform of G protein γ subunits (Botella, 2012). As no studies on *OsDEP1* in relation to heavy metal tolerance have been reported, we focused on this clone in this study.

### Confirmation that *OsDEP1* confers Cd tolerance

**Fig. 1.**
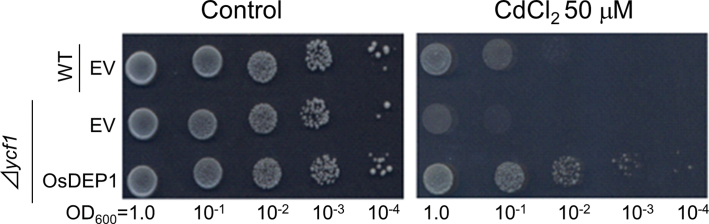
Identification of rice *OsDEP1* as a clone conferring Cd tolerance to yeast cells. Cd tolerance of yeast cells expressing *OsDEP1*. Cells of *S. cerevisiae* strain BY4742 (*Δycf1*) carrying pGK1 (EV) or pOsDEP1, and its parental strain (WT, BY4742) carrying EV were grown in SD-Ura liquid medium for 16h. The OD_600_ of the cultures was adjusted to 1.0 from which tenfold dilution series (10^–1^, 10^–2^, 10^–3^, and 10^–4^) were prepared. Subsequently, 5 μl aliquots of each dilution were spotted onto SD-Ura control medium (left panel) or medium containing 50 μM CdCl_2_ (right panel), and the cells were allowed to grow for 3 d.

The fragment covering the full-length ORF of *OsDEP1* was re-cloned into the pGK1 EV resulting in the recombinant plasmid pOsDEP1. Subsequently, pGK1 and pOsDEP1 were introduced into Cd-sensitive *Δycf1* yeast cells, and the respective transformants then spotted onto SD-Ura agar plates with or without 50 μM CdCl_2_. The *Δycf1* cells carrying pOsDEP1 grew well in Cd-containing medium, even better than the WT cells carrying EV (Fig. 1). *OsDEP1* encoded a protein composed of 426 aa. Database searches revealed that *OsDEP1* orthologues and paralogues were found in monocotyledonous plants, such as *Triticum urartu* (GenBank accession no. GQ324995; encoding a peptide of 283 aa), *Hordeum vulgare* (FJ039903; 295 aa) and *Zea mays* (NM_001158725; 408 aa), as well as in dicotyledonous plants, such as *A. thaliana* (AGG3, an isoform of γ subunits, NM_147870; 251 aa), *Glycine max* (BT095006; 209 aa), *Ricinus communis* (XM_002516219; 336 aa) and *Vitis vinefera* (CBI27799; 153 aa) (Supplementary Fig. S1 at *JXB* online). Amino acid sequence similarities between OsDEP1 and its dicotyledonous counterparts were mainly restricted to the N-terminal half (aa 40 to ~120–130) of OsDEP1.

### Impact of the C-terminal half of OsDEP1 on yeast Cd tolerance

**Fig. 2.**
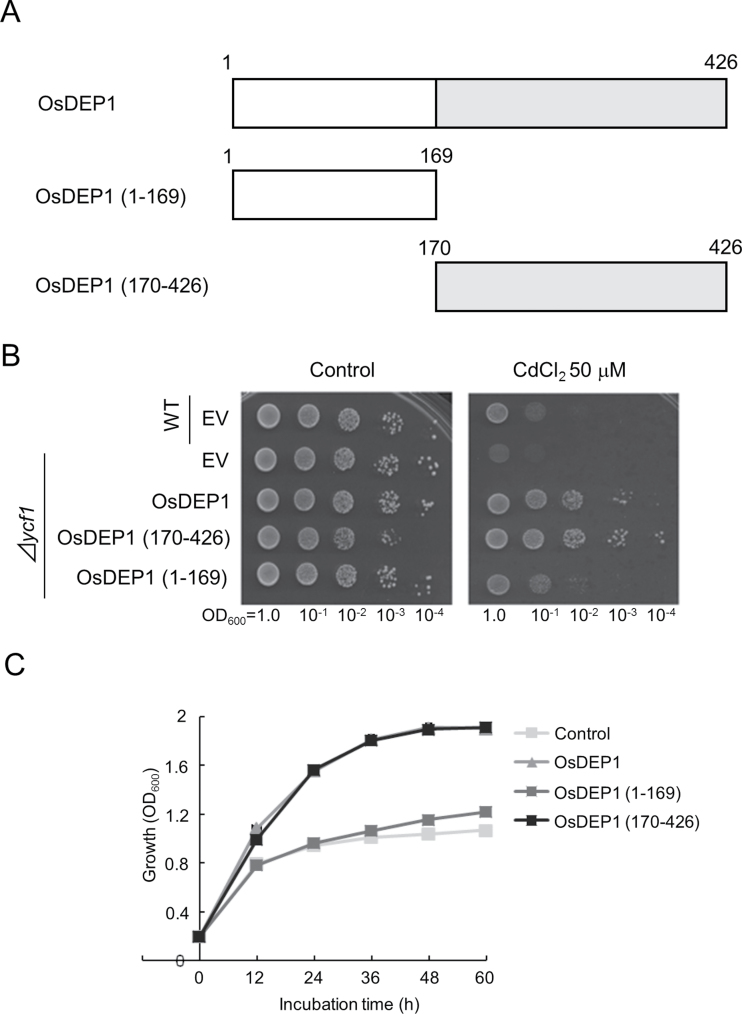
OsDEP1 is composed of two domains; the C-terminal half is Cys rich and is sufficient to confer Cd tolerance to yeast cells. (A) Schematic representation of OsDEP1 and its two truncated derivatives. OsDEP1 consists of 426 aa, of which 120 are Cys residues, with 88 of these being localized to the OsDEP1(170–426) C-terminal half. (B) Cd tolerance of yeast cells expressing intact OsDEP1 or the truncated OsDEP1(1–169) and OsDEP1(170–426) derivatives. Experiments were performed as described in Fig. 1. (C) Growth curves of yeast strains in SD-Ura liquid medium supplemented with 40 μM CdCl_2_. The growth of the yeast cells was monitored at OD_600_. The data are means ±standard deviation (SD) from three independent experiments.

OsDEP1 is composed of two domains; an N-terminal half (aa 1–169) and a C-terminal half (aa 170–426). OsDEP1 is a highly Cys-rich protein, with 120 of the 426 aa (28.2%) being Cys residues, 88 of which are localized to the C-terminal region (Fig. 2A, Supplementary Fig. S2 at *JXB* online). Two truncated derivatives of *OsDEP1*, with fragments covering the OsDEP1(1–169) and OsDEP1(170–426) regions, were cloned into the pGK1 vector resulting in constructs pOsDEP1(1–169) and pOsDEP1(170–426), respectively. These plasmids were introduced into *S. cerevisiae* BY4742 (*Δycf1*) and assayed for Cd tolerance. The results demonstrated that OsDEP1(170–426) conferred the same level of Cd tolerance to yeast cells as the intact OsDEP1 clone, whereas OsDEP1(1–169) had only a slight effect on Cd tolerance (Fig. 2B). This result was further supported by turbidity growth assays, performed in SD-Ura liquid medium containing 40 μM CdCl_2_, in which host growth was monitored after the initial turbidity of the cultures was adjusted to OD_600_=0.2. After 24h of growth, hosts carrying intact OsDEP1 or OsDEP1(170–426) reached a cell density of OD_600_=1.6, whereas hosts carrying EV or OsDEP1(1–169) only attained an OD_600_=1.0 (Fig. 2C). These results clearly demonstrated that the OsDEP1(170–426) region is necessary and sufficient to confer Cd tolerance on host yeast cells.

### Metal specificity of OsDEP1 in yeast

**Fig. 3.**
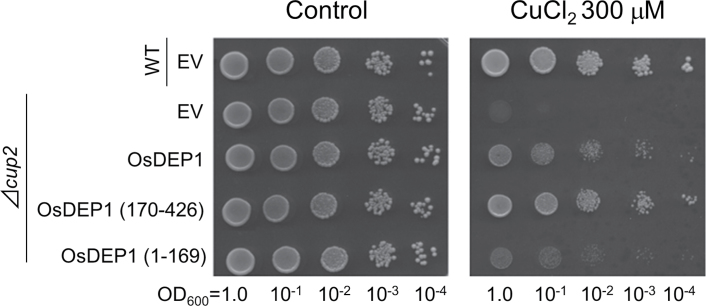
Copper tolerance of yeast cells carrying pOsDEP1 and its two deletion derivatives. Experiments were performed as described in Fig. 1 except that the 50 μM CdCl_2_ was replaced with 300 μM CuCl_2_ (right panel).

The specificity of OsDEP1-induced tolerance to specific metals in yeast was examined by an antibiotic assay method (see Supplementary Methods). The pOsDEP1 and control (EV) plasmids were introduced into the appropriate metal-sensitive yeast hosts, which were then added to molten top agarose media and immediately layered onto basal agar media. Subsequently, Cd, Cu, Co, Ni, Zn or Mn (all added as chloride salts) were infused into antibiotic assay discs and placed onto the solidified top agarose media. The diameters of the growth inhibition zones were measured after specific periods of incubation. In addition to Cd (Supplementary Fig. S3A at *JXB* online), yeast cells carrying pOsDEP1 also showed tolerance to Cu (Supplementary Fig. S3B) but not to the other four metals (Supplementary Fig. S3C–F). As the statistics did not support a significant difference in Cu response between cells (*Δcup2*) carrying EV or pOsDEP1, the Cu tolerance conferred by *OsDEP1* expression in yeast was further confirmed by dilution spot tests. In the presence of 300 μM CuCl_2,_ the *Δcup2* yeast cells carrying pOsDEP1 grew far better than those carrying EV (Fig. 3). Furthermore, as with Cd tolerance, only the C-terminal OsDEP1(170–426) region was responsible for the observed Cu tolerance phenotype, whereas the N-terminal OsDEP1(1–169) region appeared to have only a marginal effect on Cu tolerance (Fig. 3).

### Transgenic plants expressing *OsDEP1* and *OsDEP1(170–426)* are Cd tolerant, but those overexpressing *OsDEP1(1–169)* are not

**Fig. 4.**
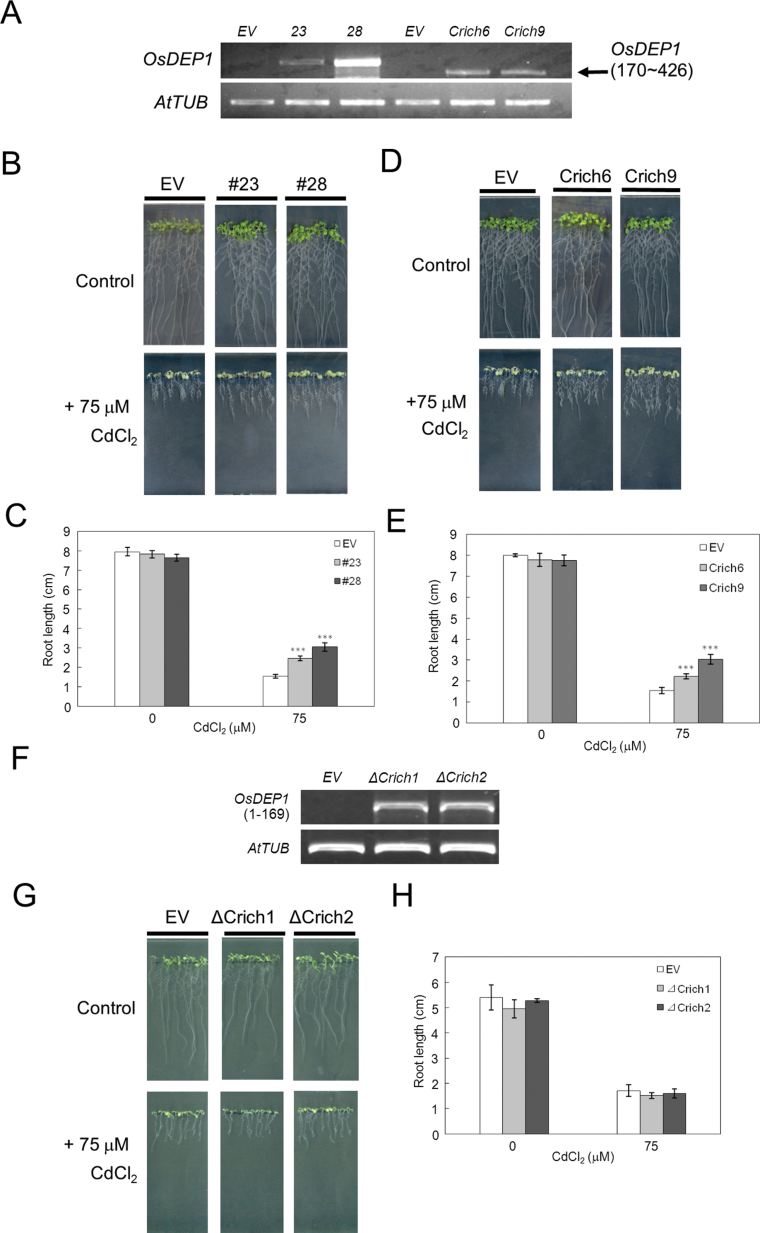
Generation of transgenic *Arabidopsis* lines expressing intact *OsDEP1* and its truncated *OsDEP1(1–169)* and *OsDEP1(170–426)* derivatives, and their responses to CdCl_2_-induced stress. (A) RT-PCR analysis of *OsDEP1* and *OsDEP1(170–426)* in each of two independent transgenic *Arabidopsis* lines. EV, control transgenic line; lines #23 and #28, transgenic lines carrying the intact *OsDEP1* gene; lines Crich6 and Crich9, transgenic lines carrying the *OsDEP1(170–426)* Cys-rich domain. The *Arabidopsis tubulin* gene (GenBank accession no. NM_001203444, *AtTUB*) was used as a loading control. (B–E) Transgenic seeds were sown onto MGRL/1% gellan gum medium with or without 75 μM CdCl_2_ for 2 weeks, at which time pictures and root length measurements were taken. (B, C) Growth responses (B) and root lengths (C) of control transgenic (EV) and two independent *OsDEP1* transgenic *Arabidopsis* lines, #23 and #28, in response to control 0 μM and 75 μM CdCl_2_. (D, E) Growth responses (D) and root lengths (E) of control transgenic (EV) and two independent transgenic *Arabidopsis* lines, Crich6 and Crich9, expressing the Cys-rich *OsDEP1*(*170–426*) region, to control (0 μM) and 75 μM CdCl_2_. Asterisks in (C) and (E) indicate that the difference from the control (EV) is statistically significant: ^***^ *P* <0.001. (F) RT-PCR analysis of *OsDEP1(1–169)* in each of two independent transgenic *Arabidopsis* lines. EV, control transgenic line; lines ΔCrich1 and ΔCrich2, transgenic lines carrying the *OsDEP1(1–169)* domain. (G, H) Growth responses (G) and root lengths (H) of control transgenic (EV) and two independent transgenic *Arabidopsis* lines, ΔCrich1 and ΔCrich2, expressing the *OsDEP1*(*1–169*) region, to control (0 μM) and 75 μM CdCl_2_. (This figure is available in colour at *JXB* online.)

To determine the role of *OsDEP1* in response to Cd *in planta*, we generated three series of *Arabidopsis* transgenic plants: those overexpressing full-length *OsDEP1* and those overexpressing its two deletion derivatives, *OsDEP1(1–169)* and *OsDEP1(170–426)*. Several independent homozygous lines expressing each of these constructs were obtained, and two lines each of the *OsDEP1* and *OsDEP1(170–426)* transgenics were selected for further study. High expression levels of *OsDEP1* in these transgenic lines were validated by RT-PCR analysis (Fig. 4A). In terms of Cd tolerance, lines #23 and #28 were tolerant to CdCl_2_ compared with control transgenic plants when tested on Cd assay plates (Fig. 4B, C). Similarly, lines Crich6 and Crich9 expressing *OsDEP1(170–426)* showed increased Cd tolerance (Fig. 4D, E). In contrast, none of the eight homozygous lines overexpressing *OsDEP1(1–169)*, including ΔCrich1 and ΔCrich2, showed any such tolerance (Fig. 4F–H).

### Transgenic plants expressing *OsDEP1* and *OsDEP1(170–426)* accumulate more Cd

**Fig. 5.**
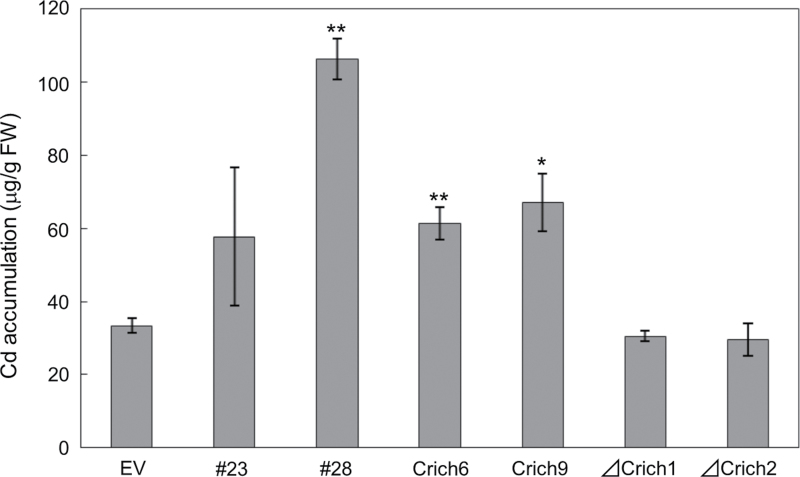
Cd content in control and transgenic *Arabidopsis* seedlings carrying intact *OsDEP1* or the truncated C-terminal *OsDEP1(170–426*) region. The control transgenics (pBI121, EV) and transgenics carrying either the intact *OsDEP1* (lines #23 and #28) or the truncated *OsDEP1*(*170–426*) region (lines Crich6 and Crich9) were sown on MGRL/1.0% gellan gum medium and then transferred to 5 μM CdCl_2_ solution for 5 d before their total Cd contents were determined. The data are means ±SD from three independent experiments. Asterisks indicate that the difference from the control (EV) was statistically significant: *P <0.05; **P <0.01.

Seedlings of the above four *Arabidopsis* lines, #23, #28, Crich6 and Crich9, together with the pBI121 vector-transformed control transgenic line (EV), were treated with 5 μM CdCl_2_ for 5 d and their total tissue Cd levels then determined. All four lines, expressing either *OsDEP1* or *OsDEP1(170–426)*, accumulated more Cd compared with the control EV and *OsDEP1(1–169)* transgenics (Fig. 5). In particular, line #28, with the highest level of transgene expression, accumulated about threefold more Cd than control transgenics (Fig. 5).

### Cd responses of loss-of-function heterotrimeric G protein gene mutants

**Fig. 6.**
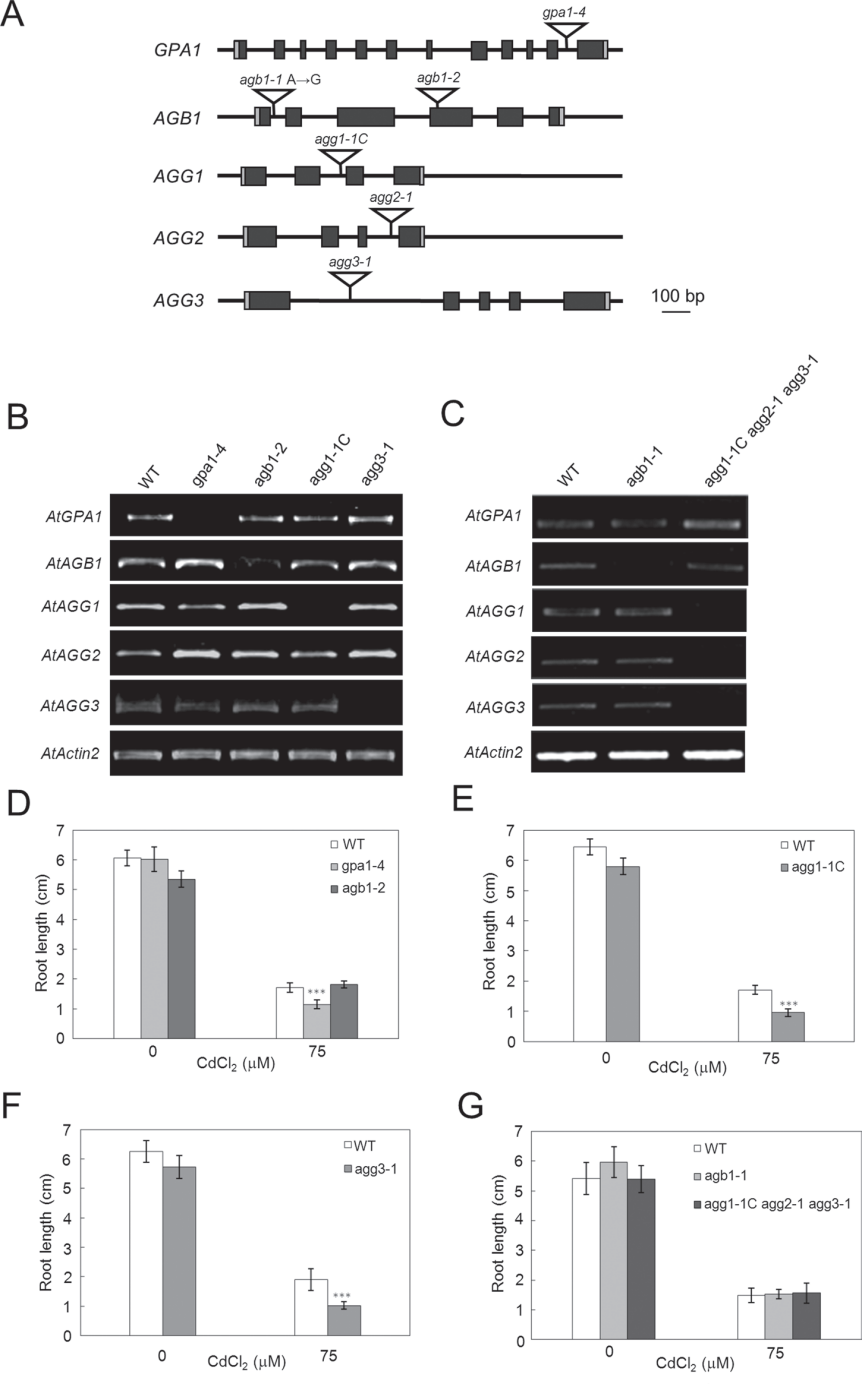
Cd response of the *Arabidopsis* mutants of G protein subunit genes. (A) Schematic of the genomic organization of *Arabidopsis* G protein subunit genes. (B, C) Confirmation of the heteromeric G protein subunit gene mutants. Expression analysis of *AtGPA1*, *AtAGB1*, *AtAGG1*, *AtAGG2*, and *AtAGG3* in WT (Col-0), *gpa1-4*, *agb1-1*, *agb1-2*, and *agg1-1C* (B), and *agg3-1* and *agg1-1C agg2-1 agg3-1* (C) *(Arabidopsis* seedlings was performed by RT-PCR using the primers listed in Supplementary Table S1 at *JXB* online. *AtActin2* was used as a loading control. (D–G) Root lengths of control WT (parental line) and mutant lines in response to control (0 μM) and 75 μM CdCl_2_: WT (Col-0) (D), Gα (*gpa1-4*) and Gβ (*agb1-2*) mutants (D); WT (Col-0) and *agg1-1C* mutant (E); WT (Col-0) and *agg3-1* mutant (F); WT (Col-0) and *agb1-1* and the triple mutant *agg1-1C agg2-1 agg3-1* (G). The data are means ±SD from three independent experiments. Asterisks indicate that the difference from the control (WT) was statistically significant: ^***^ *P* <0.001. (This figure is available in colour at *JXB* online.)

As described above, *Arabidopsis* has one α (GPA1), one β (AGB1), and three γ (AGG1, AGG2, and AGG3) subunits in its heterotrimeric G protein system (Thung *et al.*, 2012; Botella, 2012). To address whether a heterotrimeric G protein signalling pathway is involved in the Cd response, we examined the Cd sensitivity of various loss-of-function heterotrimeric G protein gene mutants of *Arabidopsis* (Fig. 6A, Supplementary Fig. S4 at *JXB* online). Validation of the mutants was tested by RT-PCR analysis (Fig. 6B, C). Two allelic mutants of *AGB1*, *agb1-1* and *agb1-2*, showed no differences in their Cd sensitivity compared with WT (Figs. 6D, G). Similarly, whereas the *agg1-1C* and *agg3-1* single mutant plants showed hypersensitivity to Cd (Fig. 6E, F), the *agg1-1C, agg2-1*, *agg3-1* triple mutant did not show any differences in its Cd sensitivity from WT plants (Fig. 6G). Based on the results of the β subunit mutant plant and triple mutant plant, we tentatively concluded that the βγ complex-mediated signalling pathway does not induce a cascade that leads to Cd tolerance. Interestingly, and in contrast, the α subunit mutant, *gpa1-4*, showed Cd hypersensitivity compared with WT lines (Fig. 6D).

## Discussion

### OsDEP1 is a useful genetic resource for phytoremediation of Cd and Cu pollution

By extensive screening of a rice cDNA library, aimed at identifying clones that could confer Cd tolerance to yeast cells, we obtained *OsDEP1*. The gene product, OsDEP1, consisted of 426 aa with a high Cys residue content (28.2%) clustered in the C-terminal half of the protein. In our studies, the Cys-rich C-terminal half (aa 170–426) of OsDEP1 was sufficient to provide exactly the same level of Cd tolerance to yeast as nascent OsDEP1, whereas the OsDEP1 N-terminal portion (aa 1–169), despite containing 32 Cys residues, could only confer limited Cd tolerance to host yeast cells (Fig. 2B, C). Similarly, in our transgenic *Arabidopsis* plants, expression of either the full-length OsDEP1 or its C-terminal Cys-rich region could provide enhanced tolerance to Cd toxicity (Fig. 4). In addition, OsDEP1 and its Cys-rich C-terminal half, but not its N-terminal half, could provide enhanced tolerance of yeast cells to Cu^2+^ but not to other heavy metals (Fig. 3, Supplementary Fig. S3). The transgenic plants expressing *OsDEP1* or *OsDEP1(170–426)* were able to accumulate more Cd in their tissues than control plants. Indeed, there was a positive correlation between the levels of *OsDEP1* or *OsDEP1(170–426)* expression and the amount of Cd accumulated (Fig. 5). Based on these results, we propose that *OsDEP1*, and even its C-terminal region alone, would be an extremely useful genetic resource for phytoremediation of Cd- and Cu-contaminated sites. Several rice cultivars are known to be Cd hyper-accumulators or Cd hypo-accumulators (Uraguchi *et al.*, 2009; Ueno *et al.*, 2011), and it will be interesting to examine whether there is any correlation between the levels and quality of *OsDEP1* transcripts in these cultivars and their ability to accumulate Cd.

### Comparison of OsDEP1 and other Cys-rich proteins involved in Cd tolerance

Several other studies have previously identified Cys-rich proteins that can provide enhanced tolerance to Cd toxicity. DcCDT1 from *D. ciliaris* is a 55 aa peptide of which 15 residues (27%) are Cys. The protein is localized to the cytoplasmic membrane and appears to function in the chelation and possible extrusion of Cd, as transgenic DcCDT1 plants accumulate considerably less Cd than controls (Kuramata *et al.*, 2009). The 25kDa CRP protein of earthworm, *Enchytraeus buchholzi*, contains 27% Cys residues that are present predominantly in a Cys–X–Cys and Cys–Cys arrangement. The gene encoding CRP is Cd inducible, suggestive of a defensive role in Cd-induced damage (Willuhn *et al.*, 1994). Similarly, OsDEP1 contains 28% Cys residues and its Cys arrangement resembles that of CRP. As with DcCDT1, the OsDEP1 protein localizes to cytoplasmic membranes and/or nuclei (Huang *et al.*, 2009; Zhou *et al.*, 2009; Taguchi-Shiobara *et al.*, 2011), but, in contrast to DcCDT1, transgenic *Arabidopsis* plants expressing *OsDEP1* or *OsDEP1*(*170–426*) accumulated more Cd than controls. Considering that OsDEP1 is a Gγ subunit, it is likely that it is localized to the inside of cytoplasmic membranes, whereas DcCDT1 may be oriented to the outside of the cytoplasmic membrane. Such a possibility would explain the observed differences in Cd uptake between the *DcCDT1*- and *OsDEP1*-expressing transgenic plants. Further work is required to substantiate this hypothesis.

### Does OsDEP1 activate the Cd tolerance system via heterotrimeric G protein signalling?

*OsDEP1* was formerly identified through quantitative trait loci analysis as a gene that controls panicle erectness, grain number per panicle and consequently grain yield (Huang *et al.*, 2009; Zhou *et al.*, 2009; Taguchi-Shiobara *et al.*, 2011). Although the functional *OsDEP1* allele was found to result in drooping panicles, deletion mutations in the Cys-rich region caused semi-dwarfism, increased spikelet numbers, and erect panicles (Huang *et al.*, 2009; Zhou *et al.*, 2009; Taguchi-Shiobara *et al.*, 2011). As reported recently, the *Arabidopsis* DEP1 homologue, AGG3, is a γ subunit of heterotrimeric G proteins, and regulates guard cell K^+^-channel activity and influences organ size and shape (Chakravorty *et al.*, 2011; Li *et al.*, 2012a,*b*). Furthermore, based on the understanding that OsDEP1 is an AGG3 homologue, and thus a G protein γ subunit, Botella (2012) discussed how the *OsDEP1* mutation could lead to increased grain yields. The N-terminal 100 aa region of OsDEP1 is a γ-domain that interacts with the β subunit of heterotrimeric G protein complexes to transduce extracellular signals via cell-surface receptors to downstream effectors (Botella, 2012). The Gβγ subunits are associated with GDP-bound Gα subunits in an inactive state. Once the extracellular portion of the G protein-coupled receptor binds its ligand, G protein signalling is activated: the activated G protein-coupled receptor triggers dissociation of the trimeric complex to Gβγ and Gα subunits, with the latter becoming activated by GTP binding. The resulting Gα and Gβγ subunits then further transmit these signals to their own effectors (Temple and Jones, 2007). As OsDEP1(170–426), which lacks the γ-domain essential for Gβ subunit association, is still able to effectively enhance Cd tolerance, it may be that OsDEP1 does not exert its Cd tolerance effect via G protein signalling.

Given that OsDEP1 is a Gγ protein, there are two possibilities for how OsDEP1 confers Cd tolerance to host plants: one is that OsDEP1 and OsDEP1(170–426) trap and detoxify these Cd ions directly, while the other possibility is that OsDEP1 activates the heterotrimeric G protein signalling pathway. Although the *Arabidopsis* AGG1 (=Gγ1) and AGG3 (=Gγ3) mutant plants were hypersensitive to Cd (Fig. 6E, F), the two allelic Gβ mutant plants and the Gγ triple mutant plant did not show any differences in root growth in the presence of Cd (Fig. 6D, G), indicating that the Gβγ pathway does not affect Cd sensitivity. On the other hand, the GPA1 (=Gα) mutant plant showed Cd hypersensitivity compared with the WT plant (Fig. 6D). As GPA1 has been implicated in guard cell K^+^-channel regulation (Wang *et al.*, 2001; Chakravorty *et al.*, 2011), it may be possible that the Gα protein signalling pathway regulates certain ion channel(s) that recognize heavy metals such as Cd and Cu ions. Of course, further study is needed to determine whether the Gα pathway is really involved in the observed Cd response. If this is the case, then functional coupling of the heterotrimeric G protein pathway to Cd responses opens a new door for exploring signal cascades in plants upon exposure to Cd or other heavy metals.

## Supplementary data

Supplementary data are available at *JXB* online.

Supplementary Fig. S1. Phylogenetic relationships between OsDEP1 and its orthologs and paralogs inother monocot and dicot plants.

Supplementary Fig. S2. Schematic representation of OsDEP1 and its amino acid sequence.

Supplementary Fig. S3. Response of yeast cells carrying pOsDEP1 to various heavy metals.

Supplementary Fig. S4. Growth responses of the loss-of-function mutants of heterotrimeric G protein(s).

Table S1. Primers used to analyse the expression of heterotrimeric G protein genes in Arabidopsis.

Supplementary Methods. (i) Yeast strain used. (ii) Metal specificity of OsDEP1 in yeast.

Supplementary Data

## Abbreviations:

ABC: ATP-binding cassette
Cd: cadmium
Cys: cysteine
EV: empty vector
Km: kanamycin
MT: metallothionein
ORF: open reading frame
RT-PCR: reverse transcription-PCR
SD: standard deviation
WT: wild type.
