# Supplementary material for: Rice DEP1, encoding a highly cysteine-rich G protein γ subunit, confers cadmium tolerance on yeast cells and plants
Source: J Exp Bot. 2013 Oct 25;64(14):4517–27. doi: 10.1093/jxb/ert267 (PMC3808331; doi:10.1093/jxb/ert267)
Supplement: Supplementary Data [file supp_64_14_4517__index.html]

Rice DEP1, encoding a highly cysteine-rich G protein γ subunit, confers cadmium tolerance on yeast cells and plants — Supplementary Data 

# Rice *DEP1*, encoding a highly cysteine-rich G protein γ subunit, confers cadmium tolerance on yeast cells and plants

## Supplementary Data

Data files

**Files in this Data Supplement:**

- Supplementary Data - Supplementary Data
