# Supplementary material for: Rice DEP1, encoding a highly cysteine-rich G protein γ subunit, confers cadmium tolerance on yeast cells and plants
Source: J Exp Bot. 2013 Oct 25;64(14):4517–27. doi: 10.1093/jxb/ert267 (PMC3808331; doi:10.1093/jxb/ert267)
Supplement: Supplementary Data [file supp_ert267_jexbot098715_file001.pdf]

Supplemental methods

Yeast strain used

Various metal-sensitive mutant strains (*Δpmr1* for Mn^{2+}, *Δzrc1* for Zn^{2+}, *Δrpd3* for Ni^{2+}, and *Δcot1* for Co^{2+}) with the following genotypes were used: *Δpmr1* (*MATα*; *his3Δ1*, *leu2Δ0*, *lys2Δ0*, *ura3Δ0*; *YGL167c::kanMX4*), *Δzrc1* (*MATα*, *his3Δ1*, *leu2Δ0*, *lys2Δ0*, *ura3Δ0*; *YMR243c::kanMX4*), *Δrpd3* (*MATα*; *his3Δ1*, *leu2Δ0*, *lys2Δ0*, *ura3Δ0*; *YNL330c::kanMX4*), and *Δcot1* (*MATα*, *his3Δ1*, *leu2Δ0*, *lys2Δ0*, *ura3Δ0*, *YOR316c::kanMX4*).

Metal specificity of OsDEP1 in yeast

To evaluate the metal specificity of *OsDEP1* in yeast, the antibiotic disc assay method was employed. In brief, the basal vector pGK1 and the pOsDEP1 construct were transformed into the respective metal-sensitive yeast strains and the resulting transformants then used for the assays. The transformed cells, grown in SD-Ura liquid media, were added to 3 ml of SD-Ura media with 0.7% molten agarose maintained at 37°C to give a cell density of OD_{600} = 0.1, then mixed quickly and poured onto 2% agar-containing SD-Ura basal medium. After solidification of the top agarose media, antibiotic assay discs (diameter 6 mm), containing specific amounts of the appropriate heavy metals, were applied. After 3 days incubation at 30°C, the diameters of the growth inhibition zones were measured.

Supplemental Table

Table S1. Primers used to analyze the expression of heterotrimeric G protein genes in Arabidopsis.

Table S1

Genes	Forward primers (5'→3')	Reverse primers(5'→3')
AtGPA1	ATGGGCTTACTCTGCAGTAG	TCATAAAAGGCCAGCCTCCA
AtAGB1	CACCATGTCTGTCTCCGAG	TCAAATCACTCTCCTGTGTGTCCTCC
AtAGG1	GCGAGAGGAAACTGTGGTTTACG	CTACTGCAGCCTTCTCCTCCATTT
AtAGG2	TCCTTGTAATTTTTGTATCCA	AGCCTCTCTCAGAGCTCACCA
AtAGG3	ATGTCTGCTCCTTCTGGCGGT	CATCTGATCTTGCAGTTGCAG

Supplemental figures

Fig. S1. Phylogenetic relationships between OsDEP1 and its orthologs and paralogs in other monocot and dicot plants with the following accession numbers: *Arabidopsis thaliana*, NM_147870 (AGG3); *Glycine max*, BT095006; *Hordeum vulgare*, FJ039903; *Oryza sativa*, Os09g0441900; *Ricinus communis*, XM_002516219; *Triticum urartu*, GQ324995; *Vitis vinifera*, CBI27799; and *Zea mays*, NM_001158725.

Fig. S2. Schematic representation of OsDEP1 and its amino acid sequence. OsDEP1 consists of a coiled-coil (CC) domain, a single transmembrane domain sandwiched between two putative nuclear localization signals (NLS), a von Willebrand factor type C module, and ends with the Cys-rich region. The Cys residues comprise 18.93% and 32.96% of the OsDEP1(1-169) and OsDEP1(170-426) regions, respectively. The Cys residues in the OsDEP1 amino acid sequence are highlighted in red.

Fig. S3. Response of yeast cells carrying pOsDEP1 to various heavy metals. The responses of yeast cells expressing pOsDEP1 to Cd, Cu, Co, Ni, Zn or Mn were evaluated by an antibiotic assay method (details as described in 'Materials and Methods'). The basal vector, pGK1 (EV), and pOsDEP1 were transformed into the respective metal-sensitive yeast strains (*Δcup2* for Cu, *Δcot1* for Co, *Δrpd3* for Ni, *Δzrc1* for Zn and *Δpmr1* for Mn) and the resulting transformants used in the antibiotic disc assay test. **A**, $CdCl_2$; **B**, $CuCl_2$; **C**, $CoCl_2$; **D**, $NiCl_2$; **E**, $ZnCl_2$; **F**, $MnCl_2$. Data are means $\pm$ SDs of three independent experiments. Asterisks indicate that the difference from the control (EV) is statistically significant. $^{***}p<0.001$.

Fig. S4. Growth responses of the loss-of-function mutants of heterotrimeric G protein(s). **A**, Growth responses of control WT (Col-0) and *agg3-1* mutant on control media (0 μM) and 75 μM $CdCl_2$-contained media. **B**, Growth responses of control WT (Col-0) and Gα (*gpa1-4*) and Gβ (*agb1-2*) mutants to control (0 μM) and 75 μM $CdCl_2$. **C**, Growth responses of WT and *agg1-1C* mutant in response to control 0 μM and 75 μM $CdCl_2$. **D**, Growth responses of WT and *agb1-1* and triple mutant *agg1-1C agg2-1*

agg3-1 to control (0 μM) and 75 μM $CdCl_2$. The experiments were repeated three times and similar results were obtained. The representative photos were shown.

Figure S1

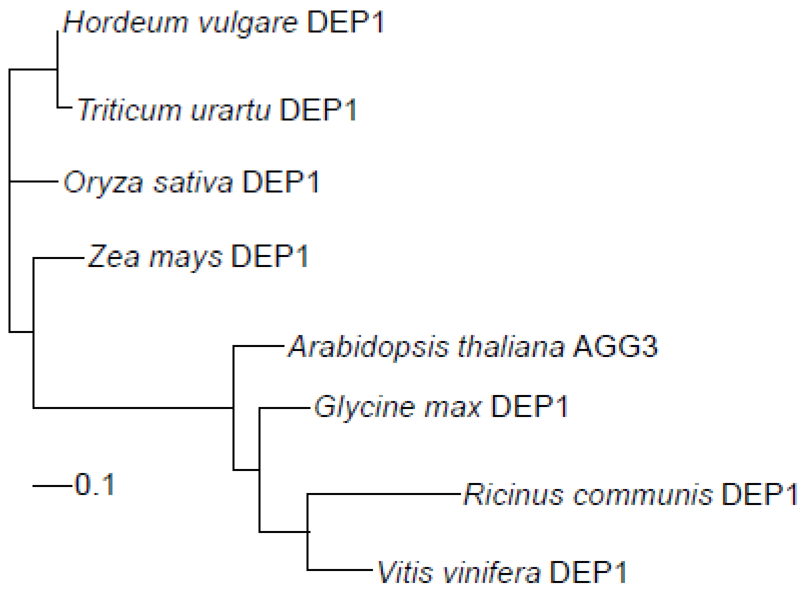

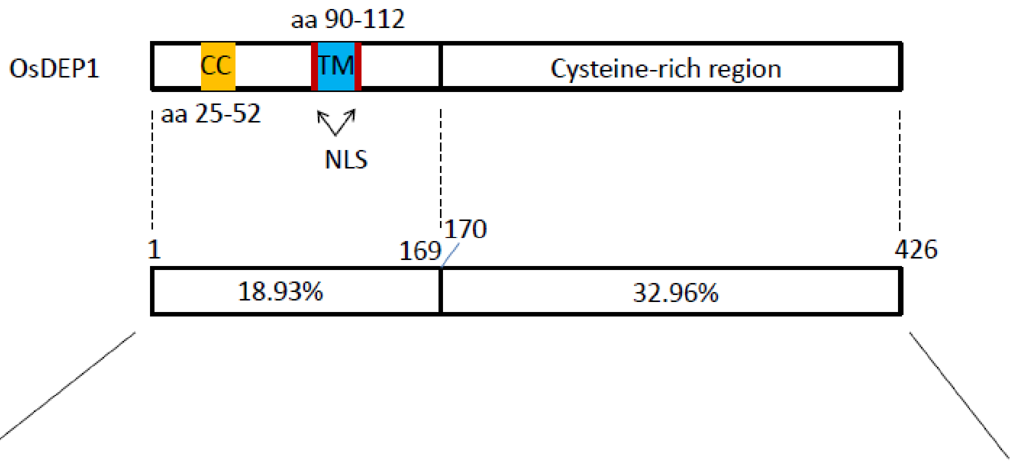


```
  1 MGEEAVVMEAPRPKSPPRYPDLCGRRRMQLEVQILSREITFLKDELHFLEGAQPVSRSGC
 61 IKEINEFVGTKHDPLIPTKRRRHRSCRLFRWIGSKLCICISCLCYCCKCSPKCKRPRCLN
121 CSCSSCCDEPCCKPNCSACCAGSCCSPDCCSCCKPNCSCCKTPSCCKPNCSCSCPSCSSC
181 CDTSCCKPSCTCFNIFSCFKSLYSCFKIPSCFKSQCNCSSPNCCTCTLPSCSCKGCACPS
241 CGCNGCGCPSCGCNGCGCPSCGCNGCGLPSCGCNGCGSCSCAQCKPDCGSCSTNCCSCKP
301 SCNGCCGEQCCRCADCFSCSCPRCSSCFNIFKCSCAGCCSSLCKCPCTTQCFSCQSSCCK
361 RQPSCCKCQSSCCEGQPSCCEGHCCSLPKPSCPECSCGCVWSCKNCTEGCRCPRCRNPCC
421 LSGCLC
```

Fig. S2

Fig S3

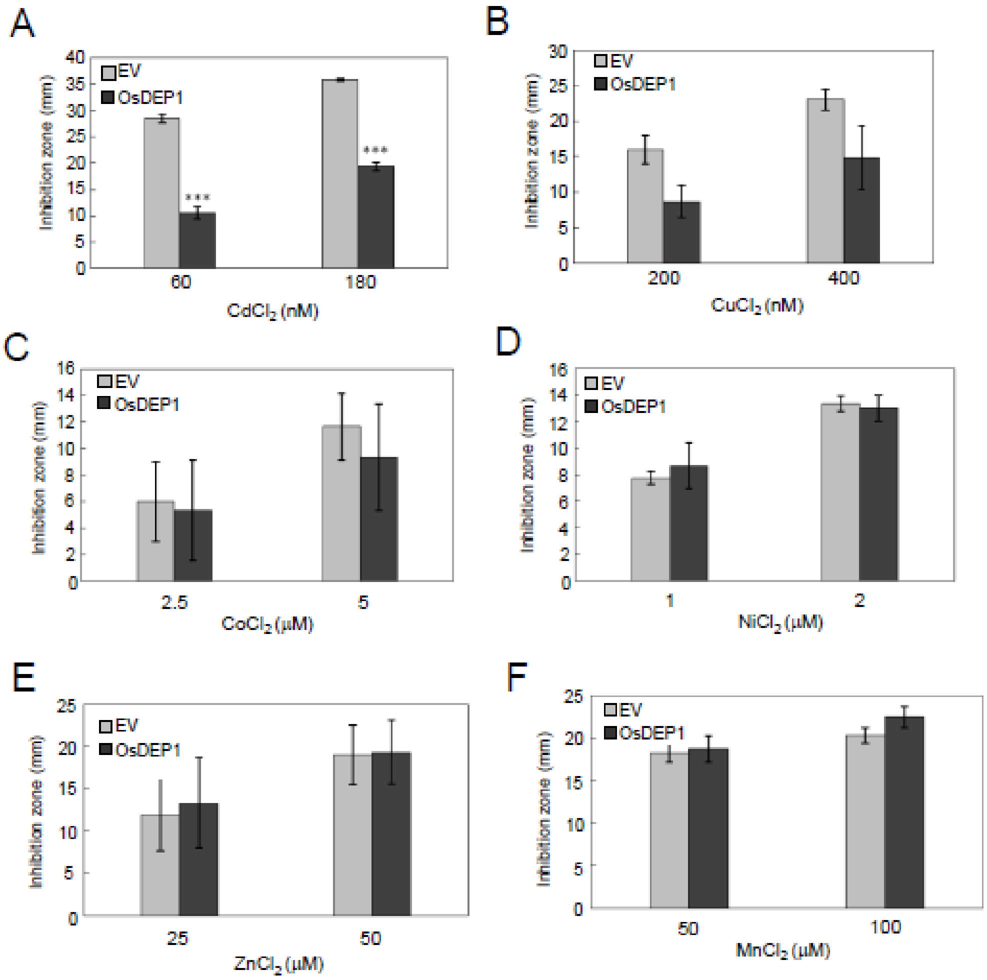
A
B
C
D
E
F
EV
OsDEP1
Inhibition zone (mm)
CdCl2 (nM)
CuCl2 (nM)
CoCl2 (µM)
NiCl2 (µM)
ZnCl2 (µM)
MnCl2 (µM)
***

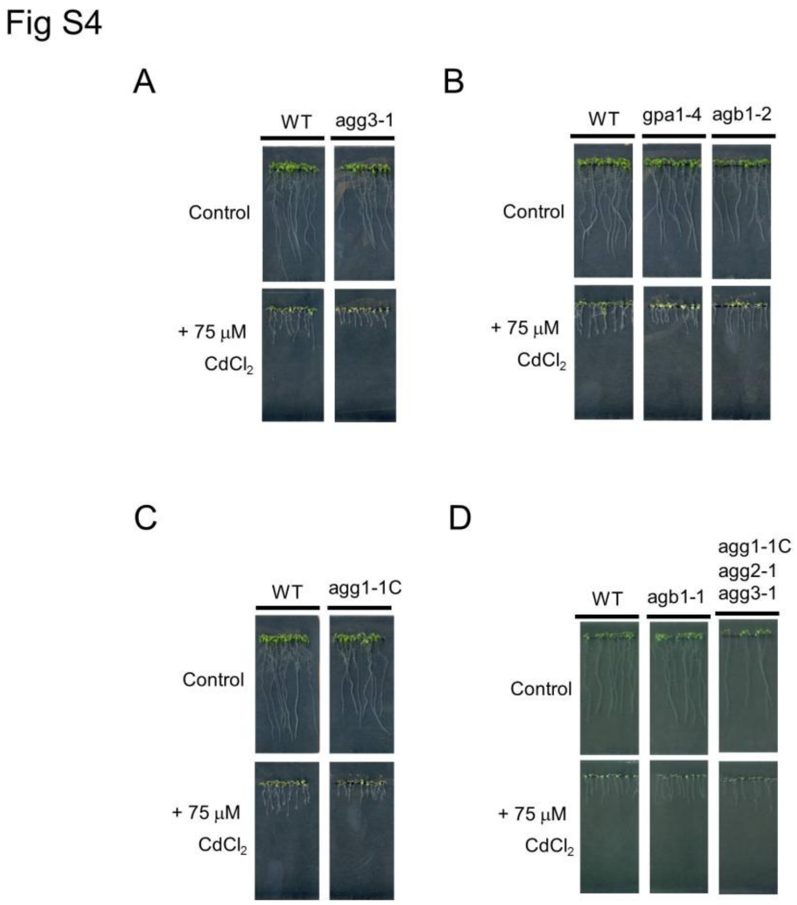
Fig S4
A
WT
agg3-1
Control
+ 75 µM
CdCl2
B
WT
gpa1-4
agb1-2
Control
+ 75 µM
CdCl2
C
WT
agg1-1C
Control
+ 75 µM
CdCl2
D
WT
agb1-1
agg1-1C
agg2-1
agg3-1
Control
+ 75 µM
CdCl2

Fig. S4.
